# Development and validation of circulating CA125 prediction models in postmenopausal women

**DOI:** 10.1186/s13048-019-0591-4

**Published:** 2019-11-26

**Authors:** Naoko Sasamoto, Ana Babic, Bernard A. Rosner, Renée T. Fortner, Allison F. Vitonis, Hidemi Yamamoto, Raina N. Fichorova, Linda J. Titus, Anne Tjønneland, Louise Hansen, Marina Kvaskoff, Agnès Fournier, Francesca Romana Mancini, Heiner Boeing, Antonia Trichopoulou, Eleni Peppa, Anna Karakatsani, Domenico Palli, Sara Grioni, Amalia Mattiello, Rosario Tumino, Valentina Fiano, N. Charlotte Onland-Moret, Elisabete Weiderpass, Inger T. Gram, J. Ramón Quirós, Leila Lujan-Barroso, Maria-Jose Sánchez, Sandra Colorado-Yohar, Aurelio Barricarte, Pilar Amiano, Annika Idahl, Eva Lundin, Hanna Sartor, Kay-Tee Khaw, Timothy J. Key, David Muller, Elio Riboli, Marc Gunter, Laure Dossus, Britton Trabert, Nicolas Wentzensen, Rudolf Kaaks, Daniel W. Cramer, Shelley S. Tworoger, Kathryn L. Terry

**Affiliations:** 1 Obstetrics and Gynecology Epidemiology Center, Brigham and Women’s Hospital and Harvard Medical School, 221 Longwood Avenue, Boston, MA 02115 USA; 2 Department of Medical Oncology, Dana-Farber Cancer Institute, Boston, MA USA; 3 Channing Division of Network Medicine, Department of Medicine, Brigham and Women’s Hospital and Harvard Medical School, Boston, MA USA; 4 Division of Cancer Epidemiology, German Cancer Research Center (DKFZ), Heidelberg, Germany; 5 Laboratory of Genital Tract Biology, Department of Obstetrics, Gynecology and Reproductive Biology, Brigham and Women’s Hospital, Boston, MA USA; 6 Departments of Epidemiology and Pediatrics, Geisel School of Medicine at Dartmouth and Norris Cotton Cancer Center, Hanover, NH USA; 7 Diet, Genes and Environment, Danish Cancer Society Research Center, Copenhagen, Denmark; 8 Department of Public Health, Faculty of Health and Medical Sciences, University of Copenhagen, Copenhagen, Denmark; 9 CESP, Fac. de médecine - Univ. Paris-Sud, Fac. de médecine - UVSQ, INSERM, Université Paris-Saclay, Villejuif, France; 10 Gustave Roussy, Villejuif, France; 11 Department of Epidemiology, German Institute of Human Nutrition Potsdam-Rehbruecke, Nuthetal, Germany; 12 Hellenic Health Foundation, Athens, Greece; 13 WHO Collaborating Center for Nutrition and Health, Unit of Nutritional Epidemiology and Nutrition in Public Health, Dept. of Hygiene, Epidemiology and Medical Statistics, School of Medicine, National and Kapodistrian University of Athens, Athens, Greece; 14 2nd Pulmonary Medicine Department, School of Medicine, “ATTIKON” University Hospital, National and Kapodistrian University of Athens, Haidari, Greece; 15 Cancer Risk Factors and Life-Style Epidemiology Unit, Institute for Cancer Research, Prevention and Clinical Network - ISPRO, Florence, Italy; 16 Epidemiology and Prevention Unit, Fondazione IRCCS Istituto Nazionale dei Tumori di Milano, Milano, Italy; 17 Dipartimento Di Medicina Clinica E Chirurgia, Federico II University, Naples, Italy; 18 Cancer Registry and Histopathology Department, “Civic - M.P. Arezzo”Hospital, ASP, Ragusa, Italy; 19 Unit of Cancer Epidemiology– CeRMS, Department of Medical Sciences, University of Turin, Turin, Italy; 20 Department of Epidemiology, Julius Center for Health Sciences and Primary Care, University Medical Center Utrecht, Utrecht University, Utrecht, Netherlands; 21 International Agency for Research on Cancer, Lyon, France; 22 Department of Community Medicine, University of Tromsø, The Arctic University of Norway, Tromsø, Norway; 23 Public Health Directorate, Astruias, Spain; 24 Unit of Nutrition and Cancer, Cancer Epidemiology Research Program, Catalan Institute of Oncology (ICO-IDIBELL), L’ Hospitalet de Llobregat, Barcelona, Spain; 25 Andalusian School of Public Health (EASP), Granada, Spain; 26 Instituto de Investigación Biosanitaria de Granada (ibs. GRANADA). Universidad de Granada, Granada, Spain; 27 CIBER of Epidemiology and Public Health (CIBERESP), Madrid, Spain; 28 Department of Epidemiology, Murcia Regional Health Council, IMIB-Arrixaca, Murcia, Spain; 29 Research Group on Demography and Health, National Faculty of Public Health, University of Antioquia, Medellín, Colombia; 30 Navarra Public Health Institute, Navarra Institute for Health Research (IdiSNA), Pamplona, Spain; 31 Public Health Division of Gipuzkoa, BioDonostia Research Institute, San Sebastian, Spain; 32 Department of Clinical Sciences, Obstetrics and Gynecology, Umeå University, Umeå, Sweden; 33 Department of Medical Biosciences, Pathology, Umeå University, Umeå, Sweden; 34 Department of Medical Imaging and Physiology, Skåne University Hospital, Lund, Sweden; 35 Department of Translational Medicine, Lund University, Lund, Sweden; 36 Cancer Epidemiology Unit, University of Cambridge, Cambridge, UK; 37 Cancer Epidemiology Unit, Nuffield Department of Population Health, University of Oxford, Oxford, UK; 38 Department of Epidemiology and Biostatistics, School of Public Health, Imperial College London, London, UK; 39 Division of Cancer Epidemiology and Genetics, National Cancer Institute, National Institutes of Health, Washington, D.C, USA; 40 Department of Cancer Epidemiology, Moffitt Cancer Center, Tampa, Florida USA; 41 Department of Epidemiology, Harvard T.H. Chan School of Public Health, Boston, MA USA

**Keywords:** Ovarian cancer, Early detection, CA125, Prediction model, Postmenopausal

## Abstract

**Background:**

Cancer Antigen 125 (CA125) is currently the best available ovarian cancer screening biomarker. However, CA125 has been limited by low sensitivity and specificity in part due to normal variation between individuals. Personal characteristics that influence CA125 could be used to improve its performance as screening biomarker.

**Methods:**

We developed and validated linear and dichotomous (≥35 U/mL) circulating CA125 prediction models in postmenopausal women without ovarian cancer who participated in one of five large population-based studies: Prostate, Lung, Colorectal, and Ovarian Cancer Screening Trial (PLCO, *n* = 26,981), European Prospective Investigation into Cancer and Nutrition (EPIC, *n* = 861), the Nurses’ Health Studies (NHS/NHSII, *n* = 81), and the New England Case Control Study (NEC, *n* = 923). The prediction models were developed using stepwise regression in PLCO and validated in EPIC, NHS/NHSII and NEC.

**Result:**

The linear CA125 prediction model, which included age, race, body mass index (BMI), smoking status and duration, parity, hysterectomy, age at menopause, and duration of hormone therapy (HT), explained 5% of the total variance of CA125. The correlation between measured and predicted CA125 was comparable in PLCO testing dataset (r = 0.18) and external validation datasets (r = 0.14). The dichotomous CA125 prediction model included age, race, BMI, smoking status and duration, hysterectomy, time since menopause, and duration of HT with AUC of 0.64 in PLCO and 0.80 in validation dataset.

**Conclusions:**

The linear prediction model explained a small portion of the total variability of CA125, suggesting the need to identify novel predictors of CA125. The dichotomous prediction model showed moderate discriminatory performance which validated well in independent dataset. Our dichotomous model could be valuable in identifying healthy women who may have elevated CA125 levels, which may contribute to reducing false positive tests using CA125 as screening biomarker.

## Background

Cancer antigen 125 (CA125) is a high molecular-weight glycoprotein (MUC16) normally expressed on tissues derived from the coelomic and Mullerian epithelial cells and aberrantly expressed on a variety of cancers, including breast, lung, leukemia, gastric, and ovarian cancer [1–3]. CA125 levels are elevated in more than 80% of ovarian cancer cases and have proven utility assessing response to therapy and prognosis [4].

While CA125 remains the most promising biomarker for ovarian cancer screening, results from two large randomized trials comparing combined CA125 and transvaginal ultrasound (TVUS) to usual care did not show significant improvement in overall survival in the screened group [5, 6]. In the United Kingdom Collaborative Trial of Ovarian Cancer Screening (UKCTOCS), stage of ovarian cancer diagnosis was earlier in the screened group, but there was no clinically significant reduction in overall mortality [6]. The Prostate, Lung, Colorectal and Ovarian Cancer Screening Trial (PLCO) showed no difference in ovarian cancer mortality between women screened with CA125 and TVUS and normal clinical care [5].

CA125 has been limited as an ovarian cancer screening biomarker by low sensitivity and specificity in part due to variation associated with differences in personal characteristics, such as age, hormone use, and menopausal status [6–10]. Identifying factors that influence CA125 levels in healthy individuals could be used to create personalized thresholds for CA125, thereby improving its performance as an ovarian cancer screening biomarker. Here we developed and validated two prediction models (linear and dichotomous) of circulating CA125 levels among postmenopausal women without ovarian cancer who had participated in one of five large population-based studies.

## Methods

### Study population

#### PLCO

The Prostate, Lung, Colorectal and Ovarian Cancer (PLCO) Screening Trial was designed to determine the efficacy of screening in reducing mortality from four mentioned cancers [11]. Briefly, from 1993 to 2001, 155,000 healthy subjects, including 78,214 women ages 55–74, were recruited from 10 study sites across the U. S and randomized to screening (the intervention arm) or usual care (the control arm). Screening intervention consisted of CA125 measurements and transvaginal ultrasound at baseline and at each of six annual screenings. For the purpose of this analysis, we used only the baseline CA125 measurements. Data on demographic and lifestyle factors were collected by questionnaires administered at baseline. Among a total of 78,214 participants, we excluded women from the control arm (*n* = 34,304), as well as those with no ovaries at baseline (*n* = 9658), a prior diagnosis of ovarian, fallopian or peritoneal cancer (*n* = 1), missing CA125 measurements at baseline (*n* = 5624), missing baseline questionnaire data (*n* = 51), a diagnosis of ovarian cancer or loss to follow-up within 3 year from baseline (*n* = 535), and those missing information on candidate predictor variables of CA125 (*n* = 1060). After these exclusions, data from 26,981 PLCO participants were available for this analysis.

#### EPIC

The European Prospective Investigation into Cancer and Nutrition (EPIC) study is a prospective cohort established between 1992 to 2000 [12]. Briefly, 519,978 participants, including 366,521 women, recruited from 23 research centers in 10 European countries, had completed questionnaires on lifestyle, medical and dietary factors. Most participants (74%) provided a blood sample at baseline. Within this cohort, a nested case-control study of ovarian cancer was designed by matching each ovarian case (*n* = 810) with up to four controls using incidence density sampling [13]. Among 1939 available controls, we defined postmenopausal women as those who met one of the following criteria at the time of blood draw: not on hormones and had not menstruated in the year prior to blood draw; on hormones and age 50 or greater; age at last menstruation was missing and age 50 or greater; had hysterectomy and age 50 or greater at the time of blood draw [7]. We excluded premenopausal women (*n* = 485), women whose menopausal status could not be determined using the algorithm above (*n* = 26), women without available CA125 measurement (*n* = 12), and those missing information on candidate predictor variables of CA125 (total *n* = 555), leading to a total study population of 861 EPIC participants for this analysis.

#### NHS/NHSII

The Nurses’ Health Study (NHS) is a prospective cohort established in 1976 when 121,700 registered nurses residing in 11 U.S. states were enrolled to investigate the long-term health outcomes of various contraceptive methods in women [14]. Nurses’ Health Study II (NHSII) is a prospective cohort established in 1989 when 116,429 nurses residing in 14 states were enrolled to study the association between oral contraceptives, diet, and lifestyle factors and long-term outcomes [15]. Participants answered baseline and biennial follow-up questionnaires about a variety of lifestyle, reproductive and medical characteristics. Blood samples were collected at two time points both in NHS (1989–1990, 2000–2002) and NHSII (1996–1999, 2010–2011). Among women with available blood samples, CA125 was measured in 152 NHS participants and 50 NHSII participants with no evidence of ovarian cancer, for a total of 202 women. We restricted to postmenopausal women defined as not having menstrual period within the past 12 months at the time of blood draw. We excluded premenopausal women (*n* = 47), those with unknown menopausal status (*n* = 14), and those missing information on candidate predictor variables of CA125 (*n* = 60), resulting in a final dataset of 81 NHS/NHSII participants for this analysis.

#### NEC

The New England Case Control Study (NEC) is a population-based ovarian cancer case-control study that enrolled participants from New Hampshire and Eastern Massachusetts over three study phases (1992–1997, 1998–2002, 2003–2008) [16]. Briefly, a total of 2075 epithelial ovarian cancer cases and 2100 controls, frequency matched on age and state of residence, participated. All the participants were interviewed in person about lifestyle factors, and medical and reproductive history. Over 95% of the study participants provided blood specimens at enrollment. Of 2100 controls, we restricted to postmenopausal women defined as: not on hormones and self-reported their menstruation had stopped or were regularly bleeding because of menopausal hormone use, were not menstruating because of hysterectomy or a medical condition/treatment and age at blood draw was 50 or greater. We excluded premenopausal women (*n* = 885), women without CA125 values (*n* = 95), and those missing information on candidate predictor variables of CA125 (*n* = 197), resulting in a total population of 923 healthy women for this analysis.

### CA125 predictor variables

Candidate predictors of CA125 were selected for this analysis based on the previously published reports [6–10]. These included age at blood draw, race, body mass index (BMI, calculated by kg/m^2^), smoking status and pack-years (calculated by number of packs of cigarettes per day multiplied by the number of smoking years), age at menarche, use of oral contraceptives (OC), parity, ovarian cysts, self-reported endometriosis, hysterectomy, age at menopause, time since menopause, hormone therapy (HT) use and duration, family history of ovarian cancer in first-degree relatives, and previous history of cancer.

We first developed the prediction models in PLCO using the candidate predictors above and then harmonized the selected final predictors across all studies so the categorization of the variables matched the variables in PLCO. Information on predictor variables listed above were collected by questionnaire data in all five studies. For PLCO, EPIC, and NEC, predictor variables and blood samples were obtained at baseline. For NHS/NHSII, age and weight were obtained from the questionnaire administered at the time of blood draw and other predictor variables were obtained from the most recent biennial questionnaire prior to the blood collection. Smoking duration among current smokers and former smokers was defined by pack-years among current and former smokers respectively across all studies. Age at menopause was defined as the self-reported age at the last menstrual period in all studies. For women who had a hysterectomy and were missing age at menopause, age at menopause was excluded. Time since menopause was calculated by subtracting age at menopause from age at blood draw.

### CA125 measurements

In PLCO, CA125 was measured using the CA-125II radioimmunoassay (Centocor) with an upper limit of normal (ULN) of 35 U/mL, described in detail elsewhere [8]. The coefficients of variation (CV) were 4.1% at a CA125 level of 52.7 U/mL, and 3.8% at a CA125 level of 106.5 U/mL [5]. In NEC and NHS/NHSII, CA125 was measured using CA-125II radioimmunoassay (Centocor) at the CERLab at Boston Children’s Hospital. The reproducibility of the assay was evaluated by including five blinded aliquots of a uniform quality control pool in each of the 46 test batches (CV = 1%). In EPIC, CA125 was measured using a volume-effective highly sensitive multiplex platform (Meso Scale Discovery, MSD) in the Genital Tract Biology Laboratory at Brigham and Women’s Hospital, with ULN of 55 U/mL. The CV for unblinded quality controls samples on each assay plates was 8.4% [13].

### Statistical analysis

CA125 levels were log-transformed to achieve normality in all of the analyses.

#### Recalibration of CA125

To account for the differences in CA125 values measured in CA125II and MSD assays, we used data from 534 NEC participants, including 353 postmenopausal women, with CA125 measured using both assays to build the recalibration model [17]. First, we built a regression model to obtain the intercept and beta coefficient (i.e. log-transformed CA125II assay value = intercept + beta*log-transformed MSD assay values). Then, we applied the intercept and beta coefficient values from this model to calculate the predicted log-transformed CA125II assay values for all the EPIC participants based on their MSD assay values. We used the predicted CA125 values based on this model for all EPIC participants in our analyses.

We calculated geometric means of CA125 values across levels of predictor variables and assessed the changes in CA125 values using percent change calculated as [exp (beta)-1] × 100 for a 1-unit change in the predictor. In order to develop the prediction model for continuous CA125 values, we randomly divided the PLCO dataset into a training (*n* = 17,987) and testing (*n* = 8994) dataset. Using the PLCO training dataset, we first examined the most appropriate way to model all the variables (continuous, categorical). For continuous variables including age, BMI, parity, and pack-years of smoking we tested for linearity of the association using restricted cubic splines [18]. For categorical variables we used likelihood ratio test to compare nested models, and Vuong test and Akaike information criterion for non-nested models [19]. Based on these evaluations, we modeled the candidate predictors as follows: age, BMI, and pack-years of smoking were modeled as continuous variables; race (white, non-white), smoking status (categorical, never, current, former), age at menarche (categorical, < 10 years, 10–11 years, 12–13 years, 14–15 years, ≥ 16 years), OC use (never, ever), parity (categorical, 0, 1, 2, 3, 4, ≥5), history of ovarian cysts (no, yes), history of endometriosis (no, yes), history of hysterectomy (no, yes), age at menopause (categorical, < 40 years, 40–44 years, 45–49 years, 50–54 years, ≥55 years), HT use (never, ever), time since menopause (categorical, < 5 years, 5–9 years, 10–14 years, 15–19 years, ≥20 years), duration of HT use (categorical, ≤ 1 years, 2–3 years, 4–5 years, 6–9 years, ≥10 years), family history of ovarian cancer (no, yes), family history of breast cancer (no, yes), and previous history of cancer (no, yes).

#### Prediction modeling

**Fig. 1:**
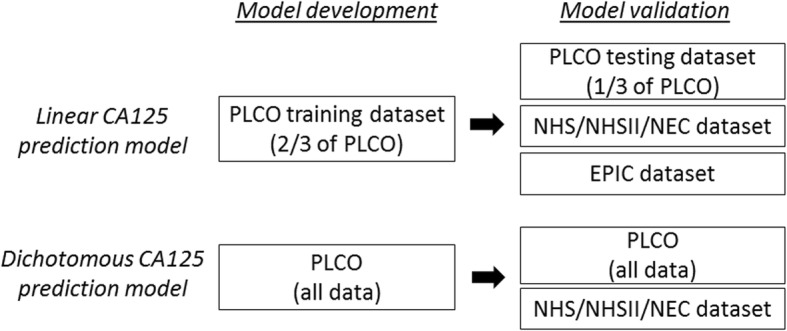
Scheme of development and validation of CA125 prediction models using five population-based studies. We developed and validated linear and dichotomous CA125 prediction models using five population-based studies: Prostate, Lung, Colorectal and Ovarian Cancer Screening Trial (PLCO), Nurses’ Health Studies (NHS/NHSII), New England Case Control Study (NEC), and European Prospective Investigation into Cancer and Nutrition (EPIC)

We developed and validated CA125 prediction models (linear and dichotomous) in postmenopausal women using five large population-based datasets (Fig. 1). We developed the prediction model in PLCO and validated the models in EPIC and in NHS/NHSII/NEC combined dataset.

#### Linear model

The association between individual predictors and CA125 levels were examined in age-adjusted models using linear regression in the entire PLCO dataset. Linear trend was tested using the continuous value of the variables (i.e. age, BMI, pack-years in current/former smokers, parity) or using the midpoint of the categories (i.e. age at menarche, duration of OC use, age at menopause, time since menopause, duration of HT use). To develop a linear CA125 prediction model, we used variables associated with CA125 at *p*-value < 0.05 in univariate analysis and performed a stepwise selection using *p*-values of 0.15 as model entry and retention criteria in the PLCO training dataset. Next, we tested the performance of the linear prediction model in PLCO testing dataset, EPIC and NHS/NHSII/NEC datasets. Briefly, predicted CA125 values in those three datasets were calculated using effect estimates from the linear prediction model developed in the PLCO training dataset and plotted against the measured CA125 values. Pearson correlation coefficient (r) was used to evaluate the linear correlation between measured and predicted CA125 values.

#### Dichotomous model

The association between individual predictors and CA125 levels ≥35 U/mL was examined in age-adjusted models using logistic regression in the entire PLCO dataset since there were only 435 participants with CA125 levels > 35 U/mL. Then, we developed a multivariate prediction model for CA125 ≥ 35 U/mL. To develop the final dichotomous CA125 prediction model, we used variables associated with CA125 levels at *p*-value ≤0.05 in the univariate analysis and performed a stepwise selection using *p*-values of 0.15 as model entry and retention criteria. Using variables selected in the stepwise selection, we evaluated the area under the curve (AUC) of receiver-operating-characteristic (ROC) curves in PLCO and NHS/NHSII/NEC datasets. EPIC was not included since only a single participant with data on all predictors had recalibrated a CA125 value ≥35 U/mL.

All statistical analyses were performed using SAS version 9.4 (SAS Institute Inc., Cary, NC).

## Results

Baseline characteristics were mostly similar across study populations, with CA125 averaging between 10 and 14 U/mL (Additional file 1: Table S1). Briefly, women were in their early 60s on average, with average BMI around 26 kg/m^2^, and mostly white race (> 90%). Approximately half of the participants reported ever smoking and most participants were parous (90%).

### Recalibration of CA125

We recalibrated the CA125 values in the EPIC participants using the model based on 534 NEC controls with CA125 measurements on both CA125II and MSD assays. The measured CA125II assay values and the recalibrated values calculated based on the recalibration model in NEC were highly correlated with Pearson correlation coefficient of 0.90 (95%CI: 0.88–0.91).

### Linear model

First, we evaluated the association between candidate predictors and continuous CA125 levels in 26,981 postmenopausal women in PLCO. Older age at blood draw, white race, lower BMI, former smoking status, shorter duration of smoking among former smokers, older age at first menstrual period, higher parity, having history of benign ovarian cyst, no history of hysterectomy, older age at last menstrual period, ever use and longer duration of hormone therapy, and shorter time since menopause were associated with higher levels of CA125 (Table 1).

**Table 1:** Age-adjusted association between selected characteristics and CA125 levels in Prostate, Lung, Colorectal and Ovarian Cancer Screening Trial (PLCO)

			Age-adjusted model	
Characteristics	N (%)	Mean CA125 (U/mL) ^a^	% change	*p*-value
Age				
< 60 years	9388 (35)	10.1 (10.0, 10.2)	Ref	Ref
60–64 years	8351 (31)	10.4 (10.3, 10.5)	3.6	<.0001
65–69 years	5814 (22)	10.7 (10.5, 10.8)	5.8	<.0001
≥70 years	3428 (13)	10.8 (10.6, 11.0)	7.1	<.0001
p-trend				<.0001
Race				
White	24,166 (90)	10.6 (10.5, 10.7)	Ref	Ref
Black	1340 (5)	8.0 (7.8, 8.3)	−24	<.0001
Hispanic	368 (1)	9.2 (8.8, 9.7)	−12.6	<.0001
Asian	922 (3)	10.0 (9.7, 10.3)	−6.4	<.0001
Pacific Islander, Native American	185 (1)	9.2 (8.6, 9.9)	−13.2	<.0001
Body mass index				
≤ 18.5 kg/m^2^	283 (1)	11.4 (10.8, 12.1)	7	0.02
18.5 < −25 kg/m^2^	10,778 (40)	10.6 (10.5, 10.7)	Ref	Ref
25 < −30 kg/m^2^	9408 (35)	10.4 (10.3, 10.5)	− 1.4	0.04
> 30 kg/m^2^	6512 (24)	10.0 (9.9, 10.1)	−5.4	<.0001
p-trend				<.0001
Smoking				
Never	15,288 (57)	10.4 (10.4, 10.5)	Ref	Ref
Current	2471 (9)	9.3 (9.1, 9.4)	−10.8	<.0001
Former	9222 (34)	10.7 (10.6, 10.8)	2.3	0.0004
Pack-years among current smokers				
< 13	49 (2)	8.5 (7.4, 9.7)	Ref	Ref
13–30	717 (29)	9.1 (8.8, 9.5)	7.4	0.33
≥ 30	1705 (69)	9.3 (9.1, 9.5)	10.1	0.18
p-trend				0.003
Pack-years among former smokers				
< 13	3370 (37)	10.5 (10.3, 10.7)	Ref	Ref
13–30	2765 (30)	10.5 (10.3, 10.7)	−0.1	0.91
≥ 30	3087 (33)	10.9 (10.7, 11.1)	3.4	0.01
p-trend				0.003
Age at first menstrual period				
< 10 years	388 (1)	9.8 (9.3, 10.3)	−5.3	0.03
10–11 years	4870 (18)	10.3 (10.1, 10.4)	−1.6	0.05
12–13 years	14,703 (54)	10.4 (10.4, 10.5)	Ref	Ref
14–15 years	5845 (22)	10.5 (10.4, 10.6)	0.1	0.85
≥ 16 years	1153 (4)	10.2 (10.0, 10.5)	−2.2	0.15
p-trend				0.18
Oral contraceptive use				
Never	12,249 (45)	10.4 (10.3, 10.5)	Ref	Ref
Ever	14,720 (55)	10.4 (10.3, 10.5)	1.0	0.11
Duration of oral contraceptive use among ever users				
< 1 year	3892 (26)	10.3 (10.2, 10.5)	Ref	Ref
2–3 years	3046 (21)	10.2 (10.1, 10.4)	−0.3	0.79
4–5 years	1989 (14)	10.2 (10.0, 10.4)	−0.7	0.62
6–9 years	2450 (17)	10.3 (10.1, 10.5)	0.7	0.58
≥ 10 years	3315 (23)	10.6 (10.4, 10.8)	2.9	0.01
p-trend				0.01
Parity				
0	2396 (9)	10.1 (9.9, 10.3)	Ref	Ref
1	1938 (7)	10.0 (9.8, 10.2)	−0.9	0.54
2	6238 (23)	10.5 (10.3, 10.6)	4.1	0.001
3	6692 (25)	10.4 (10.3, 10.6)	3.6	0.003
4	4598 (17)	10.6 (10.4, 10.7)	4.2	0.001
≥ 5	5119 (19)	10.4 (10.3, 10.6)	2.6	0.03
p-trend				0.02
Benign ovarian cyst				
No	23,561 (90)	10.4 (10.4, 10.5)	Ref	Ref
Yes	2689 (10)	10.2 (10.0, 10.4)	−2.0	0.05
Endometriosis				
No	24,587 (94)	10.4 (10.4, 10.5)	Ref	Ref
Yes	1610 (6)	10.2 (10.0, 10.4)	−1.3	0.17
Hysterectomy				
No	19,597 (73)	10.8 (10.7, 10.8)	Ref	Ref
Yes	7384 (27)	9.5 (9.4, 9.6)	−11.9	<.0001
Age at last menstrual period				
< 40 years	3655 (14)	9.4 (9.2, 9.5)	−11.8	<.0001
40–44 years	3499 (13)	10.0 (9.8, 10.2)	−6.6	<.0001
45–49 years	6092 (23)	10.4 (10.3, 10.5)	−2.9	0.0002
50–54 years	10,448 (39)	10.7 (10.6, 10.8)	Ref	Ref
≥ 55 years	3287 (12)	11.2 (11.0, 11.4)	5.3	<.0001
p-trend				<.0001
Time since menopause				
< 5 years	3254 (12)	10.6 (10.5, 10.8)	Ref	Ref
5–9 years	5120 (19)	10.5 (10.4, 10.7)	−4.9	<.0001
10–14 years	5826 (22)	10.5 (10.4, 10.6)	−8.7	<.0001
15–19 years	6356 (24)	10.2 (10.1, 10.4)	−12.9	<.0001
≥ 20 years	6425 (24)	10.2 (10.1, 10.4)	−17.4	<.0001
p-trend				<.0001
Hormone therapy use				
Never	9493 (35)	10.2 (10.1, 10.3)	Ref	Ref
Ever	17,488 (65)	10.5 (10.4, 10.6)	3.9	<.0001
Duration of hormone therapy ^b^				
≤ 1 year	3267 (19)	10.3 (10.1, 10.5)	Ref	Ref
2–3 years	2860 (16)	10.5 (10.3, 10.7)	2.9	0.03
4–5 years	2508 (14)	10.4 (10.2, 10.6)	2.3	0.08
6–9 years	3421 (20)	10.6 (10.5, 10.8)	4.3	0.001
≥ 10 years	5432 (31)	10.6 (10.4, 10.7)	1.8	0.11
p-trend				0.15
Family history of ovarian cancer				
No	25,471 (96)	10.4 (10.3, 10.5)	Ref	Ref
Yes	1049 (4)	10.3 (10.0, 10.6)	−0.8	0.59
Family history of breast cancer				
No	22,682 (86)	10.4 (10.4, 10.5)	Ref	Ref
Yes	3837 (14)	10.3 (10.1, 10.5)	−1.3	0.12
Previous history of cancer				
No	25,250 (94)	10.4 (10.3, 10.4)	Ref	Ref
Yes	1731 (6)	10.7 (10.4, 10.9)	2.3	0.06

^a^ Geometric mean (5th, 95th percentile)

^b^ Among ever hormone therapy users

We used stepwise regression analysis in the PLCO training dataset to develop the linear prediction model using variables associated with CA125 levels at *p*-value < 0.05 in univariate models (i.e. age, race, BMI, smoking status, pack-years among current smokers, pack-years among former smokers, age at first menstrual period, parity, hysterectomy, age at last menstrual period, time since menopause, and ever use and duration of HT use).

**Fig. 2:**
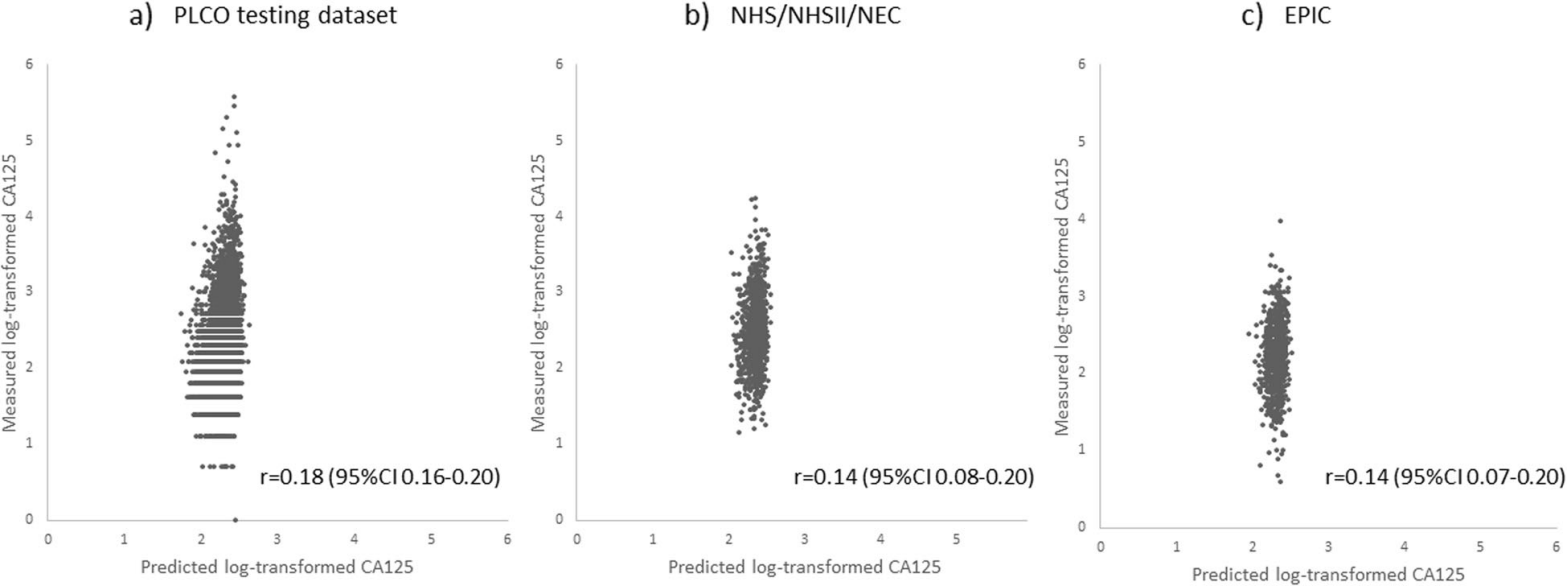
Validation of the linear CA125 prediction model in three independent datasets. The measured and the predicted log-transformed CA125 were plotted and Pearson correlation coefficients (r) were calculated to evaluate the performance of the model in three datasets. **a** Prostate, Lung, Colorectal and Ovarian Cancer Screening Trial (PLCO) testing dataset (**b**) Nurses’ Health Studies (NHS/NHSII), New England Case Control Study (NEC) (**c**) European Prospective Investigation into Cancer and Nutrition (EPIC)

The linear prediction model included age, race, BMI, smoking status, pack-years among current and former smokers, parity, hysterectomy, age at last menstrual period, and HT use and duration, which explained 5% of the variability of log-transformed CA125 (Table 2). Alternatively, when all significant predictors were included in the model without variable selection process (which consists of variables above plus age at first menstrual period and time since menopause), the r-squared was 0.05, same as that of the linear model developed using stepwise regression with fewer predictors. The associations between the selected predictors and CA125 levels in the multivariate model were similar to those observed in the univariate model. Next, we calculated the predicted log-transformed CA125 levels in the validation datasets based on the regression coefficients in the PLCO training dataset. In the PLCO testing dataset, the Pearson correlation coefficient of the measured and the predicted log-transformed CA125 was 0.18 (95%CI: 0.16–0.20) (Fig. 2a). In NHS/NHSII/NEC dataset, the Pearson correlation coefficient of the measured and the predicted log-transformed CA125 was 0.14 (95%CI: 0.08–0.20) (Fig. 2b) and in EPIC dataset it was 0.14 (95%CI: 0.07–0.20) (Fig. 2c), both similar to that in the PLCO testing dataset.

**Table 2:** Linear CA125 prediction model in Prostate, Lung, Colorectal and Ovarian Cancer Screening Trial (PLCO) training dataset

Predictor	% Change in CA125 levels ^c^	*p*-value
Age ^a^ (per year)	0.5	< 0.0001
Race		
White	Ref	Ref
Black	−20.7	< 0.0001
Hispanic	−12.0	< 0.0001
Asian	−8.5	< 0.0001
Other	−11.4	0.005
Body Mass Index ^b^ (per kg/m^2^)	−0.4	< 0.0001
Smoking status		
Never	Ref	Ref
Current	−16.9	< 0.0001
Former	0.5	0.60
Pack-years among current smokers	0.2	0.001
Pack-years among former smokers	0.1	0.02
Parity (per child)	0.9	0.0001
Hysterectomy	−11.2	< 0.0001
Age at last menstrual period		
< 40 years	−2.5	0.08
40–44 years	−1.7	0.18
45–49 years	−0.6	0.53
50–54 years	Ref	Ref
≥ 55 years	4.6	0.0001
Hormone therapy		
Never	Ref	Ref
Ever	2.1	0.85
Duration of hormone therapy		
0 years	Ref	Ref
≤ 1 year	−0.04	0.99
2–3 years	1.7	0.88
4–5 years	1.7	0.88
6–9 years	2.4	0.83
≥ 10 years	4.2	0.71
R-square	0.05	

^a^Centered at 62 years

^b^Centered at 25.9 kg/m^2^

^c^Estimate from multivariate model adjusted for all listed predictors

### Dichotomous model

We evaluated the association between candidate predictors and having CA125 ≥ 35 U/mL in PLCO (Additional file 1: Table S2). Older age at blood draw, white race, lower BMI, greater pack-years among former smokers, nulliparity, no history of hysterectomy, older age at last menstrual period, longer duration of HT use and shorter time since menopause was associated with having CA125 levels ≥35 U/mL.

**Fig. 3:**
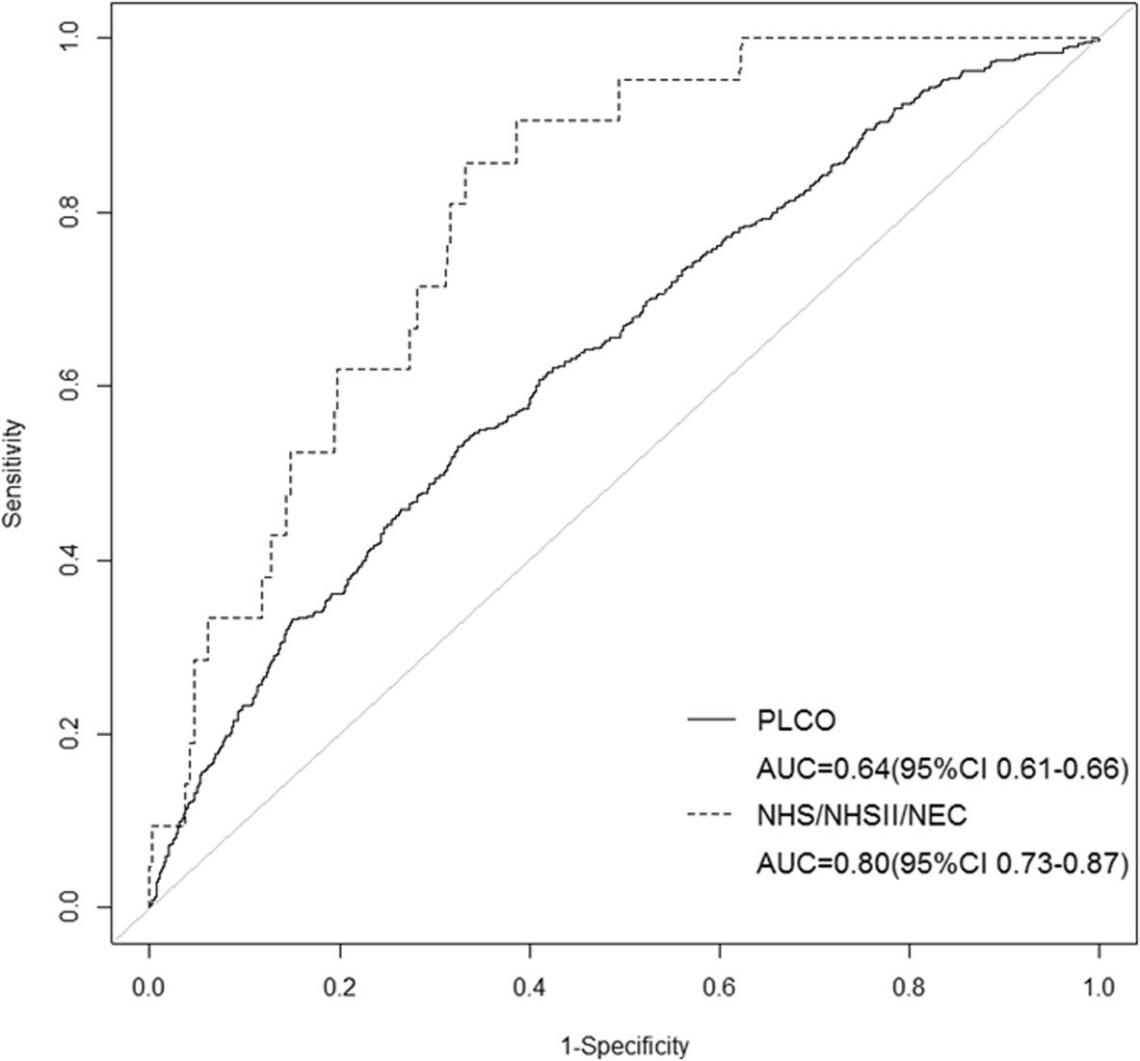
Development and validation of the dichotomous CA125 prediction model. Receiver Operating Characteristic (ROC) curves were plotted and the Area Under the Curve (AUC) were calculated to assess the discriminatory performance of the dichotomous CA125 prediction model in the Prostate, Lung, Colorectal and Ovarian Cancer Screening Trial (PLCO) (solid line) and Nurses’ Health Studies (NHS/NHSII) / New England Case Control Study (NEC) (dashed line)

We used stepwise regression analysis using all of the candidate predictors to develop the dichotomous prediction model, which included age, race, BMI, smoking status, pack-years among current and former smokers, hysterectomy, time since menopause, and duration of HT use, with an AUC of 0.64 (95%CI: 0.61–0.66) in PLCO (Table 3, Fig. 3). When we applied the regression coefficients in the PLCO to the validation dataset, the AUC was 0.80 (95%CI: 0.73–0.87) in NHS/NHSII/NEC (Fig. 3).

**Table 3:** Dichotomous CA125 prediction model in Prostate, Lung, Colorectal and Ovarian Cancer Screening Trial (PLCO)

Predictor	Odds Ratio (95% CI)^a^
Age (per year)	1.04 (1.01–1.07)
Race	
White	Ref
Non-white	0.68 (0.47–0.99)
Body mass index (kg/m^2^)	0.95 (0.93–0.97)
Smoking	
Never	Ref
Current	0.72 (0.31–1.64)
Former	1.01 (0.78–1.31)
Pack-years among current smokers	1.00 (0.98–1.02)
Pack-years among former smokers	1.01 (1.00–1.01)
Hysterectomy	
No	Ref
Yes	0.54 (0.41–0.73)
Time since menopause	
< 5 years	Ref
5–9 years	0.76 (0.52–1.04)
10–14 years	0.70 (0.46–1.05)
15–19 years	0.83 (0.52–1.31)
≥ 20 years	0.96 (0.56–1.64)
Duration of HT use	
Never users	Ref
≤ 1 year	0.60 (0.41–0.87)
2–3 years	0.80 (0.55–1.15)
4–5 years	0.88 (0.60–1.29)
6–9 years	1.27 (0.94–1.71)
≥ 10 years	1.22 (0.94–1.58)
AUC	0.64 (0.61–0.66)

^a^Mutually adjusted for all other predictors in this table

We observed that ever HT use and longer duration of use were positively associated with CA125 levels both in the linear and dichotomous model. Since women with a history of hysterectomy are more likely to have taken estrogen-only HT and type of HT may be differentially associated with CA125 levels, we conducted a stratified analysis by history of hysterectomy. However, we did not observe statistically significant effect modification by history of hysterectomy (*p*-interaction = 0.58; data not shown).

## Discussion

We confirmed factors contributing to variations in CA125 levels among postmenopausal women, including age, BMI, race, smoking status and duration, age at first menstrual period, parity, having benign ovarian cyst, hysterectomy, age at last menstrual period, HT use and duration, and time since menopause. Based on these factors, we developed and validated two prediction models in postmenopausal women without ovarian cancer using data from five large population-based studies. The final linear CA125 prediction model explained little of the total variation of CA125 values but showed similar performance in the testing and external validation datasets. The final dichotomous CA125 prediction model showed moderate discriminatory performance and validated well in the external validation dataset. Interestingly, age, BMI, race, hysterectomy, and duration of HT use were selected in both linear and dichotomous models, suggesting that these factors are robust predictors of CA125 levels in postmenopausal women.

Studies have examined personal factors that influence CA125 levels in healthy women in order to improve the clinical utility in interpreting the biomarker levels [7–10, 20]. The significant predictors selected in our linear prediction model were consistent with three prior studies that evaluated predictors of CA125 in postmenopausal women without ovarian cancer [7–9]. Older age at blood draw, non-white race, current smoking status, younger age at menopause, and history of hysterectomy were significant predictors that were consistently associated with lower CA125 levels across all of the studies that had information on these variables. Increased parity was also consistently associated with higher CA125 levels in two of the studies that assessed parity [7, 9]. HT use and longer duration were associated with higher CA125 levels in our linear prediction model, but the results were mixed in the prior two studies that examined HT use [7, 8]. This could be due to the possible differences in association by type of HT (e.g. estrogen only, estrogen and progesterone combined). If many of the hormone therapies were cyclical hormone therapies using estrogen and progesterone combined, these would result in proliferation of the endometrium and withdrawal bleeding which may possibly lead to increase in CA125 levels compared to women who are not on hormonal therapy and have no withdrawal bleeding, given that CA125 is expressed in the endometrial tissue. Although we did not observe significant effect modification of the HT associations by history of hysterectomy, given that women with history of hysterectomy are more likely to be on estrogen only HT, lack of effect modification is difficult to conclude since we were not able to evaluate the association by type of HT use due to limited information on type of HT. We did investigate former and current HT use separately, although the effect estimates were similar in these two subgroups and therefore we combined the categories into an “ever” use category when including in the final model.

In addition to examining individual predictors, we evaluated and validated the performance of the multivariate linear CA125 prediction model. Although several variables were significant predictors of CA125 in postmenopausal women and our linear prediction model was validated in independent datasets, the total variance explained by the linear prediction model was only 5%, suggesting that the known predictors may not be sufficient in explaining the CA125 variation. This is further supported by the observed lack of significant improvement in the model performance even when including all significant predictors in the model.

We also developed and validated a dichotomous prediction model using the CA125 ≥ 35 U/mL threshold. Only one prior study examined predictors of CA125 ≥ 35 U/mL in postmenopausal women, with age, BMI and hysterectomy being the only significant factors in the multivariate model [8], which were consistent with our findings. Our final dichotomous model additionally included race, smoking status and duration, time since menopause, and duration of HT use as significant predictors. Furthermore, our final dichotomous model showed moderate discriminatory performance with nine predictors which validated well in the independent dataset, suggesting the robustness of the model.

The major strength of our study was the use of data from five large population-based studies to develop and conduct internal and external validation of circulating CA125 prediction models in postmenopausal women without ovarian cancer, resulting in robust prediction models. However, there are two major limitations to the study. Since we restricted the candidate predictors to those that have been described previously in the literature, we may be lacking significant predictors which have not been investigated to date. Given that the total variance explained by our final linear model was 5%, there may be other predictors of CA125, such as genetic variants, common medications, or dietary factors, which may explain more of the variability of CA125 in postmenopausal women. Misclassification of CA125 levels in the EPIC cohort is also a concern since CA125 was measured using a different assay in this study. However, the recalibrated CA125 values based on the MSD assay values were highly correlated with the measured CA125 values using the CA125II assays in NEC (r = 0.90). In addition, the performance of the final linear model in NHS/NHSII/NEC was similar to that in EPIC, suggesting the high accuracy of the recalibration model.

## Conclusion

In summary, we developed and validated models predicting circulating CA125 in healthy postmenopausal women. The dichotomous prediction model showed moderate discriminatory performance which validated well in independent dataset. However, the linear prediction model explained a small portion of the total variability of CA125, suggesting the need to identify novel predictors of CA125. While CA125 has shown value in distinguishing malignant from benign pelvic masses [21, 22], its value as a screening biomarker in the general population has been limited by elevated levels roughly 10% of women without cancer result, which could lead to unnecessary interventions and psychological harms [23]. Our dichotomous model could be used to identify healthy women who may have CA125 levels greater than the current clinical cutoff, which may contribute to reducing false positive tests using CA125 as screening biomarker.

## Supplementary information

**Additional file 1: Table S1.** Baseline characteristics across Prostate, Lung, Colorectal and Ovarian Cancer Screening Trial (PLCO), European Prospective Investigation into Cancer and Nutrition (EPIC), Nurses’ Health Studies (NHS/NHSII), and New England Case-Control Study (NEC).**Table S2.** Age-adjusted association between predictors and CA125 levels above 35 U/mL in Prostate, Lung, Colorectal and Ovarian Cancer Screening Trial (PLCO).

## Data Availability

The datasets that support the findings of this study are available on reasonable request. The data are not publicly available due to privacy and ethical restrictions. For information on how to submit an application for gaining access to EPIC data and/or biospecimens, please follow the instructions at http://epic.iarc.fr/access/index.php.

## Abbreviations

AUC: Area under the curve
BMI: Body mass index
CA125: Cancer antigen 125
CIs: Confidence intervals
CV: Coefficients of variation
EPIC: European Prospective Investigation into Cancer and Nutrition
HT: Hormone therapy
MSD: Meso Scale Discovery
NEC: New England Case-Control Study
NHS: Nurses’ Health Studies
OC: Oral contraceptive
PLCO: Prostate, Lung, Colorectal, and Ovarian Cancer Screening Trial
ROC: Receiver-operating-characteristic
TVUS: Transvaginal ultrasound
UKCTOCS: United Kingdom Collaborative Trial of Ovarian Cancer Screening
ULN: Upper limit of normal

## References

[1] Haridas D, Ponnusamy MP, Chugh S, Lakshmanan I, Seshacharyulu P, Batra SK (2014). MUC16: molecular analysis and its functional implications in benign and malignant conditions. FASEB J.

[2] Anderson GL, McIntosh M, Wu L, Barnett M, Goodman G, Thorpe JD (2010). Assessing lead time of selected ovarian cancer biomarkers: a nested case-control study. J Natl Cancer Inst.

[3] Schorge JO, Modesitt SC, Coleman RL, Cohn DE, Kauff ND, Duska LR (2010). SGO white paper on ovarian cancer: etiology, screening and surveillance. Gynecol Oncol.

[4] Bast RC, Klug TL, St John E, Jenison E, Niloff JM, Lazarus H (1983). A radioimmunoassay using a monoclonal antibody to monitor the course of epithelial ovarian cancer. N Engl J Med.

[5] Buys SS, Partridge E, Greene MH, Prorok PC, Reding D, Riley TL (2005). Ovarian cancer screening in the prostate, lung, colorectal and ovarian (PLCO) cancer screening trial: findings from the initial screen of a randomized trial. Am J Obstet Gynecol.

[6] Jacobs IJ, Menon U, Ryan A, Gentry-Maharaj A, Burnell M, Kalsi JK (2016). Ovarian cancer screening and mortality in the UK collaborative trial of ovarian Cancer screening (UKCTOCS): a randomised controlled trial. Lancet..

[7] Fortner RT, Vitonis AF, Schock H, Husing A, Johnson T, Fichorova RN (2017). Correlates of circulating ovarian cancer early detection markers and their contribution to discrimination of early detection models: results from the EPIC cohort. J Ovarian Res.

[8] Johnson CC, Kessel B, Riley TL, Ragard LR, Williams CR, Xu JL (2008). The epidemiology of CA-125 in women without evidence of ovarian cancer in the prostate, lung, colorectal and ovarian Cancer (PLCO) screening trial. Gynecol Oncol.

[9] Pauler DK, Menon U, McIntosh M, Symecko HL, Skates SJ, Jacobs IJ (2001). Factors influencing serum CA125II levels in healthy postmenopausal women. Cancer Epidemiol Biomarkers Prev.

[10] Westhoff C, Gollub E, Patel J, Rivera H, Bast R (1990). CA 125 levels in menopausal women. Obstet Gynecol.

[11] Zhu CS, Pinsky PF, Kramer BS, Prorok PC, Purdue MP, Berg CD (2013). The prostate, lung, colorectal, and ovarian cancer screening trial and its associated research resource. J Natl Cancer Inst.

[12] Riboli E, Hunt KJ, Slimani N, Ferrari P, Norat T, Fahey M (2002). European prospective investigation into Cancer and nutrition (EPIC): study populations and data collection. Public Health Nutr.

[13] Terry KL, Schock H, Fortner RT, Husing A, Fichorova RN, Yamamoto HS (2016). A prospective evaluation of early detection biomarkers for ovarian Cancer in the European EPIC cohort. Clin Cancer Res.

[14] Colditz GA, Hankinson SE (2005). The Nurses' health study: lifestyle and health among women. Nat Rev Cancer.

[15] Rockhill B, Willett WC, Hunter DJ, Manson JE, Hankinson SE, Spiegelman D (1998). Physical activity and breast cancer risk in a cohort of young women. J Natl Cancer Inst.

[16] Terry KL, De Vivo I, Titus-Ernstoff L, Sluss PM, Cramer DW (2005). Genetic variation in the progesterone receptor gene and ovarian cancer risk. Am J Epidemiol.

[17] Eliassen AH, Hendrickson SJ, Brinton LA, Buring JE, Campos H, Dai Q (2012). Circulating carotenoids and risk of breast cancer: pooled analysis of eight prospective studies. J Natl Cancer Inst.

[18] Durrleman S, Simon R (1989). Flexible regression models with cubic splines. Stat Med.

[19] Clarke KA, Signorino CS (2010). Discriminating methods: tests for nonnested discrete choice models. Pol Stud.

[20] Lowe KA, Shah C, Wallace E, Anderson G, Paley P, McIntosh M (2008). Effects of personal characteristics on serum CA125, mesothelin, and HE4 levels in healthy postmenopausal women at high-risk for ovarian cancer. Cancer Epidemiol Biomarkers Prev.

[21] Bast RC, Skates S, Lokshin A, Moore RG (2012). Differential diagnosis of a pelvic mass: improved algorithms and novel biomarkers. Int J Gynecol Cancer.

[22] Terlikowska KM, Dobrzycka B, Witkowska AM, Mackowiak-Matejczyk B, Sledziewski TK, Kinalski M (2016). Preoperative HE4, CA125 and ROMA in the differential diagnosis of benign and malignant adnexal masses. J Ovarian Res.

[23] Force USPST, Grossman DC, Curry SJ, Owens DK, Barry MJ, Davidson KW (2018). Screening for ovarian Cancer: US preventive services task Force recommendation statement. JAMA.

