# Supplementary material for: Development and validation of circulating CA125 prediction models in postmenopausal women
Source: J Ovarian Res. 2019 Nov 26;12:116. doi: 10.1186/s13048-019-0591-4 (PMC6878636; doi:10.1186/s13048-019-0591-4)
Supplement: Supplementary file 1 — Additional file 1: Table S1. Baseline characteristics across Prostate, Lung, Colorectal and Ovarian Cancer Screening Trial (PLCO), European Prospective Investigation into Cancer and Nutrition (EPIC), Nurses’ Health Studies (NHS/NHSII), and New England Case-Control Study (NEC).Table S2. Age-adjusted association between predictors and CA125 levels above 35 U/mL in Prostate, Lung, Colorectal and Ovarian Cancer Screening Trial (PLCO). [file 13048_2019_591_MOESM1_ESM.docx]

| **Supplemental Table 1. Baseline characteristics across Prostate, Lung, Colorectal and Ovarian Cancer Screening Trial (PLCO), European Prospective Investigation into Cancer and Nutrition (EPIC), Nurses' Health Studies (NHS/NHSII), and New England Case-Control Study (NEC)** | | | | |
| --- | --- | --- | --- | --- |
| **Characteristics** | **PLCO** | **EPIC** | **NECC** | **NHS & NHSII** |
|  | **(N=26,981)** | **(N=861)** | **(N=923)** | **(N=81)** |
| ***Mean (SD)*** |  |  |  |  |
| CA125 levels (U/mL) | 11.9 (8.4) | 10.1 (4.6) | 13.5 (7.3) | 14.3 (7.2) |
| Age (years) | 62.4 (5.3) | 60.6 (5.4) | 61.1 (7.3) | 60.6 (5.9) |
| BMI (kg/m^2^) | 27.0 (5.4) | 26.6 (4.7) | 26.7 (5.7) | 26.1 (5.0) |
| Age at menarche (years) |  |  |  |  |
| ≤11 | 5,258 (20) | 117 (14) | 189 (20) | 22 (27) |
| 12 to 13 | 14,703 (55) | 348 (41) | 498 (54) | 44 (54) |
| ≥14 | 6,998 (26) | 378 (45) | 236 (26) | 15 (19) |
| Age at menopause (years) |  |  |  |  |
| <40 | 3,655 (14) | 41 (5) | 27 (3) | 0 (0) |
| 40-44 | 3,499 (13) | 105 (12) | 103 (11) | 3 (4) |
| 45-49 | 6,092 (23) | 258 (30) | 240 (26) | 22 (27) |
| 50-54 | 10,448 (39) | 377 (44) | 418 (45) | 41 (51) |
| ≥55 | 3,287 (12) | 80 (9) | 135 (15) | 15 (19) |
| Time since menopause (years) | 14.1 (7.4) | 11.8 (6.9) | 11.7 (8.1) | 11.6 (4.1) |
| Duration of hormone therapy (years)^a^ |  |  |  |  |
| ≤1 year | 3,267 (19) | 99 (35) | 58 (19) | 5 (16) |
| 2-3 years | 2,860 (16) | 62 (22) | 74 (24) | 7 (22) |
| 4-5 years | 2,508 (14) | 40 (14) | 46 (15) | 5 (16) |
| 6-9 years | 3,421 (20) | 44 (15) | 57 (18) | 2 (6) |
| ≥10 years | 5,432 (31) | 41 (14) | 75 (24) | 13 (41) |
| ***N (%)*** |  |  |  |  |
| White race | 24,166 (90) | 861 (100) | 910 (99) | 81 (100) |
| Ever smokers | 11,693 (43) | 321 (37) | 523 (57) | 39 (58) |
| Parous | 24,585 (91) | 757 (88) | 831 (90) | 77 (95) |
| Ever use of oral contraceptives | 14,720 (55) | 341 (40) | 498 (54) | 32 (40) |
| Ever use of hormone therapy | 17,488 (65) | 286 (33) | 332 (36) | 32 (40) |
| Tubal ligation | 5,855 (22) | 18 (13) | 200 (22) | 9 (11) |
| Hysterectomy | 7,384 (27) | 145 (17) | 15 (2) | 30 (37) |
| Endometriosis | 1,610 (6) | N/A | 70 (8) | 0 (0)^2^ |
| Previous history of cancer | 1,731 (6) | N/A | 127 (14) | 1 (1) |
| Family history of ovarian cancer | 1,049 (4) | N/A | 22 (2) | 1 (1) |
| ^a^ Among ever hormone therapy users | | | | |
|  |  |  |  |  |

| **Supplemental Table 2. Age-adjusted association between predictors and CA125 levels above 35 U/mL in Prostate, Lung, Colorectal and Ovarian Cancer Screening Trial (PLCO)** | | | |
| --- | --- | --- | --- |
|  | **N (%) or mean (SD)** | |  |
|  | **CA125** | **CA125** | **Age-adjusted**  **odds ratio (95 % CI) ^a^** |
|  | **<35 U/mL** | **≥35 U/mL** |  |
|  | **(N=26,546)** | **(N=435)** |  |
| **Age** ^b^ (years) | 62.3 (5.3) | 63.7 (5.5) | 1.05 (1.03-1.07) |
| **Race** |  |  |  |
| White | 23,761 (90) | 405 (93) | Ref |
| Non-white | 2,785 (10) | 30 (7) | 0.63 (0.43-0.91) |
| **Body mass index ^b^** (kg/m^2^) | 27.1 (5.4) | 25.7 (4.6) | 0.95 (0.93-0.97) |
| **Smoking** |  |  |  |
| Never | 15,046 (57) | 242 (56) | Ref |
| Current | 2,444 (9) | 27 (6) | 0.75 (0.50-1.12) |
| Former | 9,056 (34) | 166 (38) | 1.17 (0.96-1.43) |
| **Pack-years among current smokers ^b^** | 43.8 (23.6) | 44.6 (23.4) | 1.00 (0.98-1.02) |
| **Pack-years among former smokers ^b^** | 25.2 (23.5) | 28.1 (26.6) | 1.00 (1.00-1.01) |
| **Age at first menstrual period** |  |  |  |
| <10 years | 384 (1) | 4 (1) | 0.69 (0.25- 1.85) |
| 10-11 years | 4,788 (18) | 82 (19) | 1.06 (0.83- 1.37) |
| 12-13 years | 14,466 (55) | 237 (55) | Ref |
| 14-15 years | 5,750 (22) | 95 (22) | 0.98 (0.77- 1.25) |
| ≥16 years | 1,137 (4) | 16 (4) | 0.83 (0.50- 1.38) |
| **Oral contraceptive use** |  |  |  |
| Never | 12,035 (45) | 214 (49) | Ref |
| Ever | 14,500 (55) | 220 (51) | 1.04 (0.85- 1.28) |
| **Duration of oral contraceptive use ^c^** |  |  |  |
| ≤1 year | 3,824 (26) | 69 (32) | Ref |
| 2-3 years | 3,000 (21) | 46 (21) | 0.88 (0.60-1.28) |
| 4-5 years | 1,968 (14) | 21 (10) | 0.62 (0.38-1.01) |
| 6-9 years | 2,425 (17) | 25 (11) | 0.60 (0.38-0.95) |
| ≥10 years | 3,258 (23) | 58 (26) | 1.00 (0.70-1.42) |
| **Parous** |  |  |  |
| Never | 2,344 (9) | 52 (12) | Ref |
| Ever | 24,202 (91) | 383 (88) | 0.71 (0.53-0.95) |
| **Benign ovarian cyst** |  |  |  |
| No | 23,174 (90) | 387 (91) | Ref |
| Yes | 2,652 (10) | 37 (9) | 0.87 (0.62-1.23) |
| **Endometriosis** |  |  |  |
| No | 24,182 (94) | 405 (96) | Ref |
| Yes | 1,591 (6) | 19 (4) | 0.75 (0.47-1.19) |
| **Hysterectomy** |  |  |  |
| No | 19,243 (72) | 354 (81) | Ref |
| Yes | 7,303 (28) | 81 (19) | 0.60 (0.47-0.76) |
| **Age at last menstrual period** |  |  |  |
| <40 years | 3,614 (14) | 41 (9) | 0.70 (0.50- 0.99) |
| 40-44 years | 3,450 (13) | 49 (11) | 0.81 (0.59- 1.12) |
| 45-49 years | 5,983 (23) | 109 (25) | 1.04 (0.82- 1.33) |
| 50-54 years | 10,273 (39) | 175 (40) | Ref |
| ≥55 years | 3,226 (12) | 61 (14) | 1.16 (0.87- 1.56) |
| **Time since menopause** |  |  |  |
| <5 years | 3,204 (12) | 50 (11) | Ref |
| 5-9 years | 5,048 (19) | 72 (17) | 0.74 (0.51-1.08) |
| 10-14 years | 5,744 (22) | 82 (19) | 0.60 (0.41-0.89) |
| 15-19 years | 6,252 (24) | 104 (24) | 0.60 (0.40-0.91) |
| ≥20 years | 6,298 (24) | 127 (29) | 0.57 (0.36-0.91) |
| **Hormone therapy use** |  |  |  |
| Never | 9,332 (35) | 161 (37) | Ref |
| Ever | 17,214 (65) | 274 (63) | 1.00 (0.82-1.22) |
| **Duration of hormone therapy use ^d^** |  |  |  |
| ≤1 year | 3,234 (19) | 33 (12) | Ref |
| 2-3 years | 2,823 (16) | 37 (14) | 1.38 (0.86-2.21) |
| 4-5 years | 2,474 (14) | 34 (12) | 1.53 (0.94-2.48) |
| 6-9 years | 3,355 (19) | 66 (24) | 2.14 (1.40-3.27) |
| ≥10 years | 5,328 (31) | 104 (38) | 1.84 (1.24-2.73) |
| **Family history of ovarian cancer** |  |  |  |
| No | 25,061 (96) | 410 (96) | Ref |
| Yes | 1,034 (4) | 15 (4) | 0.88 (0.52-1.47) |
| **Family history of breast cancer** |  |  |  |
| No | 22,315 (86) | 367 (87) | Ref |
| Yes | 3,781 (14) | 56 (13) | 0.88 (0.66-1.17) |
| **Previous history of cancer** |  |  |  |
| No | 24,850 (94) | 400 (92) | Ref |
| Yes | 1,696 (6) | 35 (8) | 1.24 (0.87-1.75) |
| ^a^ Age-adjusted odds ratios of having CA125 levels ≥35 U/mL | | | |
| ^b^ Mean (SD) |  |  |  |
| ^c^ Among ever oral contraceptive users |  |  |  |
| ^d^ Among ever hormone therapy users |  |  |  |
